# 

**Published:** 1889-12

**Authors:** 


### BOOKS AND PAMPHLETS RECEIVED.

A Treatise on Diseases of the Nose and Throat, in two volumes. By Francke Huntington Bosworth, A. M., M. D. Volume I., Diseases of the Nose and Naso-Pharynx. New York: William Wood & Co. 1889. Price, cloth, $6.

A Text-Book of Practical Medicine. By Alfred L. Loomis, M. D., LL. D. Eighth edition. Revised and enlarged, with 215 illustrations. New York: William Wood & Co. 1889.

Hygiea Medicinsk och Farmacevtisk Manadsskrift utgifven af Svenska Läkaresällskapet. Festband Med Aledning af Tidskriftens Femtioariga Tillvaro. Stockholm: Samson & Wallin. 1889.

Massachusetts Institute of Technology. Abstract of the Proceedings of the Society of Arts, for the years from 1879–'80 to 1888–'89. Boston.

The Genu-Pectoral of Knee-Elbow Position in Obstetrics. By W. H. Wenning, M. D., Cincinnati. Reprint from Cincinnati *Lancet-Clinic*, October 12, 1889.

Ankle Injury. By J. T. Woods, M. D., Toledo, O. From Transactions Ohio State Medical Society. 1889.

Concealed Pregnancy—Its Relations to Abdominal Surgery. By Albert Vander Veer, M. D., Ph. D., of Albany, N. Y. Reprint from the *Journal of the American Medical Association*, October 19, 1889. Chicago.

The Education of Girls, from a Medical Standpoint. By Edward W. Jenks, M. D., LL. D. Reprint from Transactions of Michigan State Medical Society. 1889.

Over-Strain and Under-Power of Brain. By C. H. Hughes, M. D., St. Louis, Mo. Reprint from *Alienist and Neurologist*, October, 1889.

A Plea in Favor of Early Laparatomy for Catarrhal and Ulcerative Appendicitis, with the Report of two Cases. By N. Senn, M. D., Ph. D. Reprint from the *Journal of the American Medical Association*, November 2, 1889. Chicago.

Enteralgia and Chronic Peritonitis. By A. Jacobi, M. D., New York, N. Y.

The Early Diagnosis of Extrauterine Pregnancy. By J. M. Baldy, M. D., Philadelphia, Pa. Reprint from the *Medical Record*, September 21, 1889.

Sub-Acute or Chronic Catarrhal Pneumonia. By Herbert Judd, M. D., Galesburg, Ill.

Proceedings and Addresses at a Sanitary Convention held at Otsego, Mich., May 2 and 3, 1889. Lansing: Darius D. Throp, State Printer and Binder. 1889.

Proceedings and Addresses at a Sanitary Convention held at Tecumseh, Mich., June 6 and 7, 1889.

Cornell University, College of Agriculture. Bulletin of the Agricultural Experiment Station. Horticultural Department. X. October, 1889. Tomatoes. Published by the University, Ithaca, N. Y.

United States Department of Agriculture. Bureau of Animal Industry. Report of the United States Board of Inquiry Concerning Epizootic Diseases among Swine. Published by authority of the Secretary of Agriculture. Washington: Government Printing Office. 1889.

Announcement of the Woman's Medical College of Cincinnati. Session of 1890.

Abstract of Proceedings of the Michigan State Board of Health. Regular meeting, October 8, 1889.

State Board of Health Bulletin. Nashville, Tenn., November 20, 1889.

Health in Michigan. October, 1889.

Monthly Bulletin of the New York State Board of Health. September, 1889.

Weekly Abstract of Sanitary Reports. Volume IV., Nos. 44, 45, and 46.

---

### Literary and Other Notes.

The Johns Hopkins Hospital will issue a monthly bulletin, the first having been announced to appear in November, 1889, and nine numbers will constitute the annual issue. It will contain announcements of lecture courses, programmes of clinical and pathological study, abstracts of papers and proceedings of the medical society connected with the hospital, and reports of lectures, together with other matters of interest pertaining to the institution. The subscription price has been fixed at one dollar a year, and applications should be made to The Publication Agency of Johns Hopkins University, Baltimore, Md.

Paris Exhibition.—W. R. Warner & Co. have received a silver medal at the Paris World's Fair, being the highest of its kind, in recognition of the following claims:

*First.*—W. R. Warner & Co.'s Pills, quick solubility and accuracy.

*Second.*—Reliability and permanency unsurpassed.

*Third.*—Perfection in coating, thorough composition, and accurate subdivision.

*Fourth.*—Excellence in solubility of the finished product in from four to six minutes.

*Fifth.*—Quinine Pills, for accuracy in weight and purity of material.

Also for Warner & Co.'s Effervescent Salts.

*First.*—Superior effervescing properties.

*Second.*—General elegance and excellence.

*Third.*—Stability of the effervescing quality sustained by critical examination.

This is the thirteenth World's Fair medal which attest to their superiority. Physicians who desire these preparations should be careful to specify Warner & Co.

---

A New Journal.—Dr. I. N. Love, of St. Louis, Mo., an experienced medical writer and editor, will, we understand, soon establish a new medical journal, to be designated *The Medical Mirror.* There are not too many good medical magazines in the country, and we have a right to expect, from the reputation and experience of its editor, that the *Mirror* will take the first rank, and become a leader in the Southwest.

---

The Transactions of the American Association of Obstetricians and Gynecologists, for 1889, will be ready December 15th proximo, vide the announcement of the publisher, on page xxv. of our advertising columns.

---

The Cremation of the Dead.—Dr. William B. Clark, of Indianapolis, Ind., presents this subject in an article published in the Indianapolis *Sentinel* of September 22, 1889, in an interesting manner, showing the advantages of this method of disposing of the dead from a sanitary standpoint; also, with special reference to the prevention of infectious diseases, pointing out the danger of water pollution by the seepage of cemeteries, and burial places. It is well to disseminate such information through the popular medium of the newspaper press.

